# Advances in the mechanism of inflammasomes activation in herpes virus infection

**DOI:** 10.3389/fimmu.2024.1346878

**Published:** 2024-03-25

**Authors:** Hourui Chen, Zhijie Jian, Tong Xu, Lei Xu, Lishuang Deng, Lina Shao, Leyi Zhang, Li He, Youyou Li, Ling Zhu

**Affiliations:** ^1^ 4+4 Medical Doctor Program, Chinese Academy of Medical Sciences & Peking Union Medical College, Beijing, China; ^2^ College of Veterinary Medicine, Sichuan Agricultural University, Chengdu, China

**Keywords:** inflammasomes, herpesviruses, innate immunity, inflammatory factors, signaling pathways

## Abstract

Herpesviruses, prevalent DNA viruses with a double-stranded structure, establish enduring infections and play a part in various diseases. Despite their deployment of multiple tactics to evade the immune system, both localized and systemic inflammatory responses are triggered by the innate immune system’s recognition of them. Recent progress has offered more profound understandings of the mechanisms behind the activation of the innate immune system by herpesviruses, specifically through inflammatory signaling. This process encompasses the initiation of an intracellular nucleoprotein complex, the inflammasome associated with inflammation.Following activation, proinflammatory cytokines such as IL-1β and IL-18 are released by the inflammasome, concurrently instigating a programmed pathway for cell death. Despite the structural resemblances between herpesviruses, the distinctive methods of inflammatory activation and the ensuing outcomes in diseases linked to the virus exhibit variations.The objective of this review is to emphasize both the similarities and differences in the mechanisms of inflammatory activation among herpesviruses, elucidating their significance in diseases resulting from these viral infections.Additionally, it identifies areas requiring further research to comprehensively grasp the impact of this crucial innate immune signaling pathway on the pathogenesis of these prevalent viruses.

## Introduction

The revelation of inflammasomes has revolutionized our comprehension of the innate immune system. A sensor protein, along with a multicomponent complex comprising ASC’s caspase recruitment domain and caspase-1, forms the fundamental components of the classic inflammasome (1). The creation of this intricate structure results in the generation of pro-inflammatory cytokines, specifically interleukin IL-1β and IL-18, alongside the cleavage and initiation of gasdermin D (GSDMD). In instances where cells encounter pathogenic infections, the inflammasome’s activation can be triggered (2–4).

IL-18 and IL-1β are primarily synthesized by myeloid cells, including macrophages and dendritic cells. They play a crucial role in orchestrating immune responses against both pathogens and tissue damage (2).The induction of IL-1β serves as a vital early-stage defense mechanism employed by the host against viral and bacterial infections (5). Structurally resembling IL-1β, IL-18 primarily functions by stimulating the secretion of interferon IFN-γ from Th1 cells. Collaborating with IL-12, IL-18 fosters Th1 differentiation, thereby triggering both adaptive and innate host defense mechanisms against intracellular bacteria, viruses, and fungi (3, 6, 7).

When cells are infected by pathogens, it can induce the activation of inflammatory bodies. These entities are predominantly constituted by receptor proteins, apoptosis-associated speck-like protein containing CARD (ASC), and pro-caspase-1. The inflammasome activation leads to the initiation of caspase-1, processing and secreting mature proinflammatory cytokines such as IL-1β and IL-18, subsequently inducing cell pyroptosis (8, 9).

In 1992, the observation of rapid lytic cell death in bacterial-infected macrophages marked a significant milestone, attributed to the activity of caspase-1 (10). This phenomenon gained the term “pyroptosis” in 2001, representing a lytic form of programmed cell death. Pyroptosis in mammalian cells is widely recognized to rely on gasdermins, a family of pore-forming proteins (11). his family encompasses GSDMA, GSDMB, GSDMC, GSDMD, GSDME, and GSDMF (PJVK/DFNB59). The identification of GSDMD as a downstream effector of the inflammasome in 2015 further solidified the understanding of pyroptotic mechanisms (12). dditionally, inflammasome-independent mechanisms activate other members of the gasdermin family, such as GSDMA and GSDMB (13). Despite lacking the GSDMD cleavage sequence and being non-substrates for caspase-11, the expression of the N-terminal domain of all gasdermins induced pyroptosis in HEK293T cells (14).

The host’s immune defense system identifies viral genomes and various pathogenic agents, including pathogen-associated molecular patterns (PAMPs), using pattern recognition receptors (PRRs). This immediate defense mechanism is activated by PRRs recognition, impacting the adaptive immune response (15). PRRs encompass toll-like receptors (TLRs), retinoic acid-inducible gene-like receptors (RLRs), nucleotide-binding domain-like receptors (NLRs), and AIM2-like receptors (ALRs). TLRs can respond to various ligands. Their activation leads to the stimulation of nuclear factor-kappa B (NF-κB) and interferon regulatory factor 3/7 (IRF3/7). The signaling of IRF3/7 initiates the production of type I interferons (IFNs) and pro-inflammatory cytokines, which include pro-IL-1β (16). The nuclear translocation of NF-κB results in the gene transcription vital for inflammasome signaling, encompassing pro-IL-1β, pro-IL-18, and pro-caspase-1 (17). It’s noteworthy that the initiation of several inflammasomes doesn’t indispensably depend on this initial activation step (18, 19). The activation phase, as the second step, necessitates the sensor protein’s recognition of its corresponding signal. This leads to ASC oligomerization, inflammasome assembly, and the cleavage of pro-IL-1β and pro-IL-18 by caspase-1. Multiple sensor proteins can initiate this activation step, detecting various PAMPs and danger-associated molecular patterns (DAMPs). Typically, these sensor proteins belong to the NLR family, like NLRP1, NLRC4, and NLPR3 (20, 21). For instance, the activation of the NLRP3 inflammasome by herpes simplex virus 1 (HSV-1/HHV-1), and the activation of the AIM2 inflammasome (17) by cytomegalovirus (CMV/HHV-5) (20–22). Studies indicate that AIM2 can discern the intricate structure of bacteria, viruses, and even the host’s double-stranded DNA (dsDNA). This recognition, in turn, triggers downstream inflammatory signaling pathways (23, 24). Upon recognition of viral DNA by AIM2, it has the capability to enlist the adaptor protein ASC, forming an inflammasome in conjunction with caspase-1. The activated caspase-1 precisely cleaves pro-IL-1β and pro-IL-18, resulting in the secretion of mature IL-1β and IL-18, respectively (25). This activation of caspase-1 initiates the cleavage of pro-IL-1β and pro-IL-18, leading to the subsequent release of mature IL-1β and IL-18.

This review will concentrate on the canonical inflammasomes dependent on caspase-1. Besides the cleavage of IL-1β and IL-18, caspase-1 also induces the cleavage of GSDMD. Subsequently, GSDMD creates pores in the plasma membrane, causing cell death through pyroptosis and facilitating the release of IL-1β and IL-18 (26).

## Herpes virus is a common pathogen

Herpesviruses represent prevalent pathogens in the human population, capable of inducing a variety of illnesses, spanning from unnoticed infections to afflictions such as tumorigenesis, retinitis, and fatal encephalitis. The virions of Human Herpesviruses (HHVs) showcase a capsid with an icosahedral structure, enveloping a genome consisting of double-stranded DNA. Surrounding the capsid is a protein layer known as the coat, devoid of structure, and an outer envelope comprising a lipid bilayer decorated with glycoproteins. Classified into three subfamilies—α-, β-, and γ-herpesviruses—HHVs possess the unique ability to establish latent infections that endure throughout an individual’s lifetime (27). Herpesvirus A comprises HSV-1 and HSV-2. Although the majority of immunocompetent individuals undergo a mild, self-limiting illness after HSV infection, it may result in diverse conditions like cold sores, genital herpes, herpes stromal keratitis, eczema herpeticum, disseminated disease in newborns, meningitis, and herpes simplex encephalitis (28). Despite displaying a wide cell tropism, these viruses lay dormant in ganglia along the neural axis until reactivation transpires, leading to the recurrence of viral shedding or the manifestation of the disease (27). HSV-1 and HSV-2 exhibit high prevalence rates in adults, infecting over half of the population with either or both viruses (29–31). CMV, HHV-6A, HHV-6B, and HHV-7 belong to the herpes β viruses. These viral entities possess the capability to establish latent infections in lymphocytes and other hematopoietic cells (32). In the United States, cytomegalovirus infects approximately 40 to 60% of individuals by adulthood, attaining almost 100% seroprevalence in certain global regions (33, 34). Among them, CMV holds clinical significance, emerging as the primary culprit behind neonatal complications and occurrences in immunosuppressed populations (32, 35, 36). Encephalitis caused by acute HHV-6 may stem from inherent immune system anomalies related to the virus. Isolated acute HHV-6 infection can lead to encephalitis in individuals with inherited primary immunodeficiencies, especially those with autosomal recessive (AR) partial IRAK-4 deficiency. The manifestation of severe viral diseases, notably HHV-6 encephalitis upon acute infection, characterizes AR IRAK-4 deficiency (37).

The γ Herpesvirus subfamily includes the EBV/HHV-4 and KSHV/HHV-8. Worldwide, EBV affects 70% to 95% of adults, typically acquired during childhood. It predominantly dwells in memory B cells. Although initial infection frequently shows no symptoms, it has the potential to induce mononucleosis in adolescents and adults. This condition is characterized by manifestations such as fever, malaise, myalgia, pharyngitis, palatal petechiae, cervical lymphadenopathy, splenomegaly, and atypical lymphocytosis (38).BV is also correlated with numerous malignant tumors, and EBV is associated with diverse malignancies, including nasopharyngeal carcinoma (NPC) and Burkitt lymphoma (BL) (27, 39, 40). Varied expression of latent EBV genes in oral SCC, ranging from 15% to 70%, has been reported, but the role of EBV in oral squamous cell carcinoma (SCC) remains uncertain (41, 42). KSHV displays higher seroprevalence in sub-Saharan Africa (30%-50%) and the Mediterranean region. It serves as a prevalent pathogen in AIDS-related malignant tumors, such as Kaposi’s sarcoma (KS), primary effusion lymphoma (PEL), multicenter Castleman’s disease (MCD), and KSHV inflammatory cytokine syndrome (KICS). These conditions predominantly impact individuals with compromised immune function (43, 44).

## The impact of inflammasome activation varies in diseases caused by herpesviruses

Inflammasome activation is a prevalent occurrence during viral infections. Throughout the viral infection progression, it participates in recognizing innate immunity and initiating inflammatory responses. Recent studies have documented instances of inflammasome activation in infections induced by influenza virus, hepatitis C virus, human immunodeficiency virus (HIV), and herpesviruses (45–48). Subsequently, we shall delineate the influence of inflammasome activation in the course of alphaherpesvirus infections.

Data derived from mouse models of HSV-1 and HSV-2 infection strongly affirm the vital function of inflammasome activation in averting and alleviating diseases. Mice with an IL-1β knockout (KO) exhibit markedly increased susceptibility to lethal HSV-1 encephalitis when contrasted with wild-type (WT) mice (49). This emphasizes the paramount significance of IL-1β, generated by monocytes/macrophages in the early stages of infection, in providing protection against excessive disease manifestation (50). Similarly, research indicates the advantageous role of IL-18 in activating Natural Killer (NK) cells, offering defense against fatal HSV-1 pneumonia and HSV-2 genital disease (51–55). Furthermore, IL-18 potentially aids in ameliorating ocular lesions associated with herpetic stromal keratitis (HSK) (56). In a study utilizing the mouse HSK model, it was observed that mice lacking NLRP3 (NLRP3 KO) exhibit more pronounced HSK lesions compared to their wild-type (WT) counterparts (57). Research also suggests that HSV-1 initiates Gasdermin D-dependent pyroptosis through the activation of NLRP3 inflammasomes in microglial cells in mice, resulting in the generation of IL-1β and caspase-1. The suppression of HSV-1-induced Gasdermin D-dependent pyroptosis can be achieved by inhibiting the activation of NLRP3 inflammasomes in microglial cells (58).

Nevertheless, certain investigations propose that inflammasome activation might be harmful to the host during HSV-1/HSV-2 infections. For instance, the presence of IL-1β has the potential to stimulate HSV-1 proliferation in neurons (59). In a mouse model of herpetic keratitis, highly virulent HSV-1 induces a stronger inflammatory response associated with severe corneal lesions. The virulence of HSV-1 is implicated in the synchronized early induction of NLRP3, NLRP12, and IFI16 inflammasomes, leading to destructive inflammatory responses, which are associated with increased cleavage of Caspase-1, IL-1β, and IL-18 (60). In a model of encephalitis, HSV-1 infection led to decreased inflammation and lower mortality in mice lacking ASC and NLRP3, as opposed to the WT mice (61). Furthermore, in a model of genital infection, the pathology of HSV-2 is linked to IL-18 (62). These findings indicate varied inflammasome activation roles in distinct diseases, possibly associated with the disease nature, its progression, or other undisclosed factors.

Limited documentation exists regarding inflammasome activation in the context of VZV infection. The initiation of NLRP3 inflammasome assembly and the production of IL-1β by VZV occur in diverse human cell lines that facilitate VZV replication. In a model involving skin xenografts in severe combined immunodeficiency (SCID) mice, VZV prompts the activation of the NLRP3 inflammasome (63). In a VZV-related postherpetic neuralgia (PHN) rat model, diminishing the secretion of IL-1β and IL-18 proves advantageous in alleviating PHN. This implies that the activation of the inflammasome may inflict harm in the context of PHN (64). Nevertheless, the question of whether inflammasome activation benefits the host in VZV infection or is essential for efficient virus dissemination remains uncertain.

The impact of inflammasome activation amid CMV infection remains somewhat ambiguous. HCMV demonstrates the capability to invade nearly all organs and cells within the body, residing within arterial smooth muscle cells and vascular endothelial cells. This presence results in vascular lesions linked to diverse cardiovascular diseases. In a particular investigation, it was observed that CMV infection induces the upregulation of ETAR by suppressing the expression of miRNA-1929-3p in the host. This, in turn, triggers the activation of the NLRP3 inflammasome, fostering the proliferation of vascular smooth muscle cells and contributing to the initiation and progression of hypertension (65). Consequently, these findings indicate a pivotal role of NLRP3 in cardiovascular diseases induced by CMV.

HHV6 encompasses two distinct viruses, HHV6A and HHV6B, prevalent in the human population. A limited-scale investigation did discern a potential association between the copy number of HHV-6 and the levels of IL-1β in children experiencing febrile convulsions. Earlier research has demonstrated the detection of HHV-6 DNA in the bloodstream of a minor fraction of patients undergoing febrile convulsions. Furthermore, elevated IL-1β concentrations are observable in the saliva of convulsing children. Notably, the copy number of HHV-6 exhibits a positive correlation with IL-1β levels in saliva (66). It is widely believed that systemic HHV6 infection with high levels of HHV6 and viremia can induce acute myocarditis. In another study reported, mild myocarditis was not associated with the presence of low levels of HHV6 DNA, and the NLRP3 pathway did not appear to be modulated (67). Herpes simplex virus type 1 (HSV-1) infects more than 50% of the global population, and infection of the cornea with HSV-1 can lead to subclinical inflammation, which can develop into mild epithelial herpetic keratitis, or it can spread deeper in the corneal stroma and develop into more severe inflammatory disease. In coulon et al., expression levels of NLRP3, NLRP12, and IFI16 inflammatory bodies were associated with severe corneal inflammatory herpes disease (68).

Concerning the activation of inflammasomes in EBV-related illnesses, the onset of EBV-induced infectious mononucleosis results in an upsurge of IL-18 levels in plasma and a substantial increase in IL-18 within lymphoid tissue (69). Similarly, children experiencing acute EBV infection exhibit elevated IL-1β levels in their tonsils (70). These initial investigations propose a correlation between acute EBV infection and inflammasome-driven cytokines within the body. In a recent examination, tumor cells positive for EBV demonstrated a high expression of HMGB1 and sustained the presence of the EBV-dissolving switch ZEBRA through NLRP3. This mechanism facilitated the replication and release of virions (71). Furthermore, the connection between the activation of the EBV replication switch and EBV PTLD, mediated by diabetes-associated inflammatory bodies, is evident in these correlations (72).

Substantial evidence indicates the activation of inflammasomes in diseases induced by KSHV. The herpes virus KSHV is linked to Kaposi’s sarcoma, characterized as an angioplastic tumor formation that necessitates a consistent IL-1β environment. Functioning as a cytoplasmic sensor for foreign molecules, the inflammasome can independently trigger caspase-1 activation and the maturation of IL-1β cytokine. This, in turn, establishes a stable environment conducive to the development of angioplastic tumors (73). Earlier research has also identified elevated IL-1β levels in KS patients, and when introduced to cultured KS cells, IL-1β actively promotes tumorigenesis (74, 75). In summary, these findings suggest that inflammasome activation seems to promote tumorgenesis. In addition, these discoveries imply that inflammasome activation appears to contribute to tumorigenesis. Additionally, during the latent infection phase of KSHV, the activation of inflammatory bodies and IL-1β inhibit the reactivation of KSHV from latency, favoring the incubation of KSHV (73). **Table 1** summarizes the effects of various inflammasome activations on herpes virus-associated disease.

**Table 1:** the effects of various inflammasome activations on herpes virus-associated disease.

virus	Effect on clinical disease	reference
HSV-1	IL-1β has a protective effect against encephalitis caused by HSV-1.	(49, 50)
HSV-1/HSV-2	IL-18 facilitates the activation of natural killer cells and provides protection against fatal HSV-1 pneumonia and HSV-2 genital disease.	(51–55)
	IL-1β induces the proliferation of HSV-1 in neurons, leading to more severe herpetic keratitis in mice.	(59, 60)
	In a model of encephalitis, HSV-1 infection led to decreased inflammation and lower mortality in mice lacking ASC and NLRP3, as opposed to the WT mice.	(61)
VZV	Reducing the release of IL-1β and IL-18 was beneficial for improving post-herpes zoster neuralgia in mice infected with vzv.	(64)
CMV	CMV infection can activate the NLRP3 inflammasome and promote the occurrence and development of hypertension.	(65)
HHV6	IL-1β was positively correlated with HHV-6 copy number in saliva of children with convulsion.	(66)
EBV	Ebv-induced infectious mononucleosis results in elevated levels of IL-18 in plasma and maintains the expression of EBV-dissolving switch ZEBRA via NLRP3, thus promoting the replication and release of virions.	(69–72)
KSHV	Inflammasome activation and IL-1β can inhibit the reactivation of KSHV from the incubation period, promote the incubation period of KSHV, and promote the development of tumors.	(73–75)

## Infection with herpesviruses can trigger multiple inflammasomes

In herpesvirus pathogenesis, the pivotal roles of IL-1β and IL-18 necessitated the use of additional methods to illustrate the direct activation of the inflammasome by HSV-1. Early investigations into the AIM2 inflammasome unveiled that HSV-1 triggers its activation in macrophages, independently of the dsDNA sensor (76). Subsequently, the viral protein VP22 was identified as a specific inhibitor of the AIM2 inflammasome during HSV-1 infection (77), suggesting the participation of alternative sensors in HSV-1 infection. Notably, NLRP3 consistently takes center stage in HSV-1 inflammasome activation, as observed in keratinocytes (78), human foreskin fibroblasts (HFFs) (22), and macrophages (79). The mechanism through which HSV-1 activates NLRP3 involves the stimulator of interferon genes (STING), which recruits NLRP3 to the endoplasmic reticulum, initiating inflammasome activation by modulating K48- and K63-linked polyubiquitination (80). The activation of other inflammasomes by HSV-1 is contingent upon the specific infection models employed.

The proposed role of the AIM2 inflammasome involves functioning in keratinocyte infection and specific mouse models (60, 81). In HFFs, the sensing of HSV-1 by IFI16 is thought to trigger inflammasome activation. However, neither AIM2 nor IFI16 is deemed necessary for inflammasome activation in macrophages (63, 82). During HSV-1 infection in mice, proteins like NLRP12 experience upregulation, yet their essentiality for or direct participation in HSV-1-induced inflammasome activation remains uncertain (83). Although NLRP3 appears to be the primary inflammasome activated in HSV-1 infection, there is a possibility of other inflammasomes being activated in specific cell types or tissues.

Aside from the acknowledged AIM2-inhibitory role of VP22, it remains uncertain whether HSV-1 encompasses additional elements for inflammasome inhibition or regulation. ICP0 is believed to diminish the induction of NLRP3 and IFI16 in HFFs, while replication-dependent factors might impede NLRP3 inflammasome activation in macrophages (84, 85). The direct influence of inflammasome activation on HSV-1 replication is not clear, creating an open field of investigation into viral regulation of inflammasome activation.

Limited research has delved into the mechanism of VZV-induced inflammasome activation. Data indicates its activation of the NLRP3 inflammasome in at least three cell types permissive for VZV replication *in vitro* (86).

Though CMV is recognized for robustly triggering innate immune signaling, recent discoveries indicate that CMV’s activation of the inflammasome relies on AIM2 and is bolstered by STING (87). *In vitro*, the growth of CMV is impeded by IL-1β, and CMV’s immediate early 86 kDa protein (IE-86) restrains the release of IL-1β from cells infected by CMV (88). This underscores the vital significance of inflammasome regulation during CMV infection, influencing both the viral life cycle and pathogenesis.

EBV infection induces the elevation of IFI16 and NLRP3 inflammasomes in both primary and latent infections, confirming their activation by EBV and subsequent facilitation of IL-1β maturation. IFI16, an innate immune sensor situated in the nucleus and irrespective of DNA sequence, detects the nuclear replication process of EBV within infected nuclei (73, 89). Upon recognition, it forms an inflammasome complex with ASC and pre-caspase-1, initiating the synthesis of IL-1β and IL-18. Additionally, infection with Herpes simplex virus (HSV) also instigates the creation of NLRP3 inflammasomes and consequent IL-1β production in human TH-1 cells, fibroblasts, and melanoma cells (61). Remarkably, AIM2 does not contribute to NLRP3 recruitment, underscoring distinct secretion pathways for various inflammasome types. In the absence of AIM2, EBV infection activates the NLRP3 inflammasome complex through caspase-1 activation, fostering the maturation of IL-1β and IL-18 (81, 90).

Macrophages play a crucial role in PRV replication, acting as the primary source of proinflammatory cytokines. Past studies have revealed that PRV infection initiates GSDMD-dependent pyroptosis through the assembly of two inflammasomes: the NLRP3 inflammasome and IFI16 inflammasome. This process is characterized by the release of lactate dehydrogenase (LDH) and the secretion of IL-1β (91). Viral proteins, including SARS-CoV-2 N and E proteins, can activate the NLRP3 inflammasome, leading to excessive inflammation. This implies that viral replication or protein production is vital for PRV-induced inflammatory responses, as it triggers the NLRP3 inflammasome and contributes to cell death in PRV-infected 3D4/21 cells. The activation of the NLRP3 inflammasome is also observed in the brains of mice infected with PRV, resulting in the formation of the NLRP3-ASC-CasP1 complex. To further investigate this process, we will establish a porcine NLRP3 inflammasome system by transfecting plasmids encoding the three components of the inflammasome (NLRP3, ASC, CASP1), along with the pro-IL-1β substrate (92). In summary, PRV infection triggers both NLRP3 inflammasome activation and IL-1β secretion.

Bovine herpesvirus 1 (BoHV-1) is a viral pathogen that induces inflammation in cattle by infiltrating and inflaming tissues. In the course of acute infection, two essential components for inflammasome formation, namely the DNA sensor IFI16 and NLRP3, are triggered in bovine kidney cells. IFI16 can be identified in punctate particles within the cytoplasm and nucleus (93). During productive infection, there is a notable surge in the number of cells exhibiting positive results for caspase 1, an enzyme activated subsequent to inflammasome formation. These discoveries indicate that BoHV-1 infection instigates inflammasome formation and furnishes proof of caspase 1 activation. However, the influence of caspase 1 on CRIB cell-induced infection is not substantial, underscoring the necessity for further research to comprehend the mechanism of BoHV-1-induced inflammasome.

KSHV has been discovered to harbor DNA and transcripts in various human cell types, including B cells, endothelial cells, epithelial cells, macrophages, and keratinocytes. In the course of KSHV infection, the inflammasome’s activation necessitates IFI16 and results in the conversion of pre-IL-1β into active IL-1β (94). The expression of IFI16 in endothelial cells correlates with ASC, a crucial participant in inflammasome assembly. The process involves the oligomerization and pre-recruitment of caspase-1 through ASC upon the recognition of diverse stimuli by sensor proteins. Caspase-1 self-cleavage leads to the formation of active caspase-1 p10/p20 tetramers. Following activation, caspase-1 cleaves the inactive pre-forms of IL-1β and IL-18, releasing these cytokines (90). The indispensable role of IFI16 or ASC in virus-induced caspase-1 processing was demonstrated using short hairpin RNA (shRNA) targeting them. Previously, IFI16 was not regarded as an inflammasome activator due to its inability to effectively activate the inflammasome when overexpressed, in contrast to AIM2 function (91). IFI16 expression in endothelial cells is associated with ASC which plays a crucial role in the assembly of inflammasomes. Inflammasome assembly involves the oligomerization and pre-recruitment of caspase-1 through ASC upon recognition of various stimuli by sensor proteins. The self-cleavage of caspase-1 leads to the production of active caspase-1 p10/p20 tetramers. Subsequently, activated caspase-1 cleaves the inactive pre-forms of IL-1β and IL-18 to secrete these cytokines (95). The essential role of IFI16 or ASC in virus-induced caspase-1 processing was demonstrated using short hairpin RNA (shRNA) targeting them.Previously, IFI16 was not considered as an inflammasome activator due to its inability to constructively activate the inflammasome when overexpressed, unlike AIM2 function (77). The activation of the AIM2 inflammasome is triggered by DNA, resulting in caspase-1 activation and the release of pro-inflammatory cytokines IL-1β and IL-18, which play crucial roles in the host’s innate immune response against various pathogens. Despite some viruses employing strategies to counteract the inflammasome-mediated induction of pro-inflammatory cytokines, their relevance *in vivo* remains unclear. Polymorphisms in regulatory proteins within the IL-18 pathway, including IL-18 receptor and IL-18 receptor helper proteins, have been reported to be associated with positive HSV-1, HSV-2, and human cytomegalovirus seropositivity (73, 96). However, the intricate interactions between KSHV and inflammasome responses have not been fully elucidated yet.

## Conclusions

The current data emphasizes the importance of the interaction between herpesviruses and inflammasome signaling. This interaction influences not only the viral life cycle but also the development of diseases associated with herpesviruses. However, it is evident that the mechanism of inflammasome activation and its consequences on the host are distinct for each herpesvirus, potentially varying within specific infected cells or tissues. Consequently, findings from studies on one herpesvirus cannot be extrapolated to other members of the herpesvirus family and must be approached with caution when considering other infection models for the same virus. Further exploration into the implications of inflammasome activation on diseases induced by herpesviruses, as well as the ways in which herpesviruses trigger and regulate inflammasomes, is imperative. This is especially crucial as inflammasome modulators progress through clinical trials. The open question remains whether these therapeutics can enhance herpesvirus-related diseases or potentially exacerbate these pathologies. HHV infections are widely prevalent, and it is still crucial for the scientific community to thoroughly investigate their impact on herpesvirus-related diseases before the extensive application of inflammasome modulators.

## Author contributions

HC: Writing – original draft. ZJ: Writing – review & editing. TX: Writing – review & editing. LX: Writing – review & editing. LD: Writing – review & editing. LS: Writing – review & editing. LeZ: Writing – review & editing. LH: Writing – review & editing. YL: Writing – review & editing. LiZ: Writing – review & editing.
